# Cellular, molecular, and metabolic aspects of developing lungs in congenital diaphragmatic hernia

**DOI:** 10.3389/fped.2022.932463

**Published:** 2022-11-15

**Authors:** Shahana Perveen, Marta Frigeni, Helene Benveniste, Dalibor Kurepa

**Affiliations:** ^1^ Department Pediatrics, Feinstein Institute for Medical Research, New York, NY, United States; ^2^ Department of pediatrics, Donald and Barbara Zucker School of Medicine at Hofstra-Northwell, Hempstead, NY, United States; ^3^ Department Pediatrics/Neonatal Perinatal Medicine, Cohen Children's Medical Center, New Hyde Park, NY, United States; ^4^ School of Medicine, Yale University, New Haven, CT, United States

**Keywords:** congenital diaphragmatic hermia, lung developement, fetus, pulmonary hyperetnsion, new born

## Introduction

Congenital diaphragmatic hernia (CDH) is a complex birth anomaly described in medical literature since the early 18th century. It is a defect in the diaphragm that leads to herniation of the abdominal contents into the thoracic cavity. As a result, the growth of the developing lung and vasculature is impaired, and respiratory failure can occur at birth with devastating consequences. Infants are born with CDH every 10 min worldwide and 1 in 2500–3500 babies are diagnosed with CDH every year in the USA (1). It is associated with 8% of all major congenital anomalies and accounts for >1% of total infant mortality in the USA. The estimated cost of CDH management in the USA is >230 million dollars.

CDH associated lung hypoplasia and abnormal pulmonary vascular malformation, including defective angiogenesis leading to persistent pulmonary hypertension in newborns (PPHN) are the main cause of high mortality and morbidity. Despite advances in neonatal care and evolving new modalities of treatment CDH remains a challenging problem (2). Surgical CDH repair after birth is not always linked with improved survival (3). In addition, infants with CDH usually show limited response to vasodilator therapy due to extensive vascular remodeling (4).

## Normal embryological development of the diaphragm

The diaphragm is a sheet of muscles separating the abdominal cavity from the thoracic cavity. The diaphragm begins to develop at approximately 4 weeks of gestation and is fully formed by 12 weeks of gestation. It develops from the septum transversum, pleuroperitoneal folds, and somite adjacent to the neural tube as a source of diaphragm muscles and derivatives of dorsal mesentery. The septum transversum is a thick mass of cranial mesenchyme, formed in the embryo, and gives rise to parts of the thoracic diaphragm and the ventral mesentery of the foregut. The septum transversum develops around 22 days gestation rostral to the developing heart. During normal development, the pleuroperitoneal folds fuse with the septum transversum, the esophageal mesentery, and the muscular ingrowth from the body wall invades the folds, forming the muscular part of the diaphragm.

## Pathogenesis of CDH

CDH is a multifactorial disease with poorly understood etiology and pathogenesis. Genetic factors, environmental exposure, and nutritional deficiencies may play a role in the development of this complex disease. The embryological basis of CDH propose that the defect happens secondary to the failure of parts of the diaphragm to fuse resulting in a patent pleuroperitoneal canal, thereby inducing CDH. Another theory, known as “the dual hit phenomenon” describes lung hypoplasia (primary hit) as the primary causal factor in the pathogenesis of CDH; this is followed by abdominal content herniation (secondary hit) that further compromises the growth of an already hypoplastic lung (5). In addition, diverse genetic or environmental factors can cause disruption of mesenchymal cell function in the primordial diaphragm as well as thoracic organs including lungs and heart. An additional hypothesis postulates that an imbalance between cell apoptosis and cell proliferation plays a role in the pathogenesis of CDH; however, analysis of the affected patient's tissues did not demonstrate increased apoptosis, thus making this hypothesis less likely (6). Irrespective of the underlying cellular mechanisms, the defect in the diaphragm causes the abdominal viscera to herniate into the thoracic cavity. This process ultimately results in abnormal lung and vascular development as well as increased vasoreactivity, manifesting as persistent pulmonary hypertension at birth.

## Classification of CDH

The most common classifications of CDH are isolated or non-syndromic vs. complex or syndromic CDH. CDH can be classified based on location, size, organs involved, the extent of lung involvement, and the presence of associated abnormalities. Posterolateral, Bochdalek type accounts for approximately 70%–75% of cases, anterior, Morgagni type CDH for 27%. Central Hernia—Septum Transversum type CDH for 2%–3%. The majority of cases are posterolateral, Bochdalek type CDH on the left side (85%), while 13% of cases occur on the right side, and only 2% are bilateral (7).

**Figure 1:**
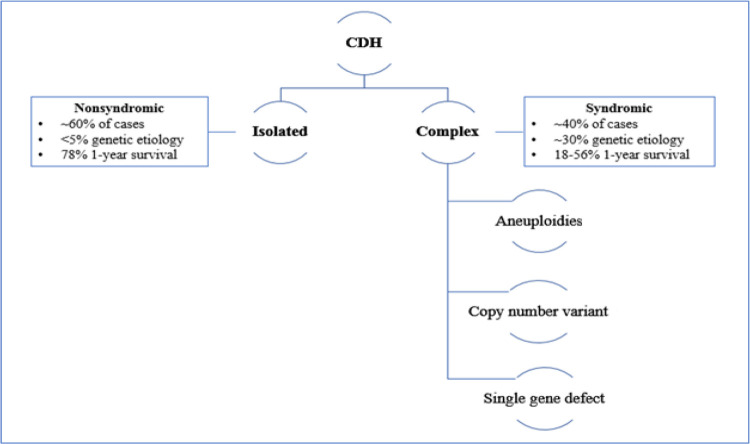
Classification of CDH.

The presence of additional anomalies is more common in the complex type and it directly influences morbidity and mortality (8) (Figure 1).

Isolated cases of CDH are associated with better survival: while the presence of additional anomalies in the complex type of CDH directly influences the patient's morbidity and mortality (8). (Figure 1) The presence of liver herniation has been described as a poor prognostic factor, with a 45% survival rate compared to 74% in patients without liver herniation. In addition, liver herniation has been associated with high predictivity of ECMO treatment: 80% of the patient with liver herniation required ECMO compared to only 25% without liver herniation (9).

Lung to Head Ratio (LHR) is an ultrasound based calculation first described in 1996, as a prenatal predictor of mortality for fetuses with CDH. An LHR <1 is associated with a poor prognosis (10). As LHR did not account for gestational age initially, it is now expressed as a percentage of what can be expected in a normal fetus or observed-to-expected (O/E) LHR which is independent of gestational age. LHR along with the presence of liver herniation is the most often used predictor of clinical outcome.

MRI-derived lung volume metrics measuring lung size, and degree of pulmonary hypoplasia, have also been found to predict mortality and pulmonary morbidity, as well as chronic lung disease (11).

Infants with CDH shows significant high survival with O/E-TFLV > 35% compared to those <35% (94% survival vs. 56% survival) (12).

The exact etiology of differences in the severity of CDH and associated mortality and morbidity remains unclear, however, the presence or absence of different risk factors plays important role in the overall outcome of CDH. Risk factors like congenital anomalies, side of the defect (right side vs. left side, 33.3% vs. 7.8%,), position of the liver, intrathoracic position associated with higher mortality (22.2% vs. 3.7%), presence of pulmonary hypertension, and need for extracorporeal membrane oxygenation(ECMO) contribute to poor outcome (13) The survival in patients with an isolated CDH was no different from those with another associated congenital anomaly although there was a trend toward improved survival with isolated CDH (80.2% vs. 70.2%). Presence of complex congenital heart disease and genetic disorder in combination with CDH has been described with increased mortality (14).

## Embryological basis of CDH

Impaired fusion of the diaphragm's embryological components may produce any of the following: hernia of Morgagni, a pleuroperitoneal defect, a hiatal hernia, or eventration of the diaphragm (15). Pleuroperitoneal folds (PPF) are the major source of the diaphragm's muscle connective tissue and central tendon (16). Defects in the development of the PPFs will lead to incomplete development of the diaphragm. Several genes associated with CDH are expressed in relation to PPFs and may be the cause of incomplete diaphragmatic development (17, 18). The genetic inactivation of *GATA*-4 in mice showed the development of localized regions that lack muscle progenitors leading to amuscular patches in the developing diaphragm that are mechanically weaker resulting in herniation. Failure of migration of muscle progenitors from the somites into the PPFs can lead to a muscle-less diaphragm or hemidiaphragm (19). In summary, based on the data described above the general view holds that CDH arises from the primary defects in PPFs secondary to defective generation and migration of cells or muscularization of the diaphragm (20). Genetic defects in septum transversum have also been associated with CDH in mice models (21).

## Cellular aspect of CDH

The mesothelium is an outer tissue layer covering the thoracic organs including the heart, diaphragm, and the lung (22). Lung mesothelium plays an important role in the early stages of lung development through expression of key genes, such as retinoic acid dehydrogenase (*ALDH*1*a*2) and fibroblast growth factor 9 (*Fgf*9) (23). The mesothelium processes retinoic acid from Vitamin A *via* retinaldehyde dehydrogenase 2 (*RALDH*2 also known as *ALDH*1*A*2) and has been linked to CDH in humans as well as mouse models of CDH (24, 25). Supporting the concept of essential Vitamin A signaling in the diaphragm development, both pathogenic variants in and copy number variations (*CNVs*) of genes involved in Vitamin A metabolism and signaling pathway were described affecting several levels of retinoid signaling (26). Retinoid signaling (*RA*) is critical for both diaphragm and lung development, and disruptions and dysregulation of this pathway could contribute to isolated CDH etiology (24). Similarly, Wilms's tumor 1 (*WT*1) is a Wilms tumor suppressor gene and a transcription factor expressed in the muscular diaphragm, pleural and abdominal mesothelium including the heart and kidney. *WT*1 has been identified as an important defective gene expressed in the mesothelium and is thought to be responsible for CDH in human and animal models (27).

Pathogenic variants in the *FOG*2 gene, a transcriptional co-regulator, have been linked to CDH and pulmonary hypoplasia in human and mice models. The pathogenic variants of *GATA*-4 gene, a transcription factor known to functionally interact with *FOG*2, have also been described as an important factor for proper mesenchymal cell function in developing diaphragm, heart, and lung (28). In summary, mesenchymal cell function is an important aspect of proper diaphragm, heart, and lung development and many geneses including *GATA*-4, *FOG*2, 1 may play a role but the exact mechanistic pathway and association remain unclear. It is crucial to understand the effect of pathogenic variants in these genes in the development of the normal diaphragm and CDH, as well as their possible role in the development of the heart and lungs. CDH associated genes are known to have pleiotropic effects and expressivity that varies between affected individuals (29).

Along with lung hypoplasia, CDH is associated with significant pulmonary hypertension attributed to the early disturbance in vascular growth and pulmonary vascular remodeling (30). As vascular development precedes tissue growth, abnormal cellular proliferation and interactions have been proposed as an underlying mechanism. Increased proliferation of vascular smooth muscle cells (SMCs) and abnormal signaling of pulmonary arterial endothelial cells (PAECs) has also been reported. Defective signaling of PAECs leads to increased proliferation of pulmonary SMCs with subsequent development of pulmonary hypertension (31). Endothelium −1 is a potent vasoconstrictor and plays an important role in vascular remolding associated with CDH. ET-1 is a member of a peptide family that is secreted from endothelial cells. The upregulated expression of ET-1 and ETA receptor mRNA has been correlated with pulmonary vasoconstriction and altered pulmonary vascular muscularization in CDH (32). ET1 acts as a vascular smooth muscle cell mitogen *via* the production of reactive oxygen species (ROS) and decreases pulmonary nitric oxide (NO) bioavailability. ET1 levels are highly associated with disease severity in infants with CDH (33). Rho and the Rho kinase pathway play an important role in various cellular functions, including smooth muscle contraction. Rho kinase activity has been found to play an important role in the acute pulmonary vasoconstrictor response to several different stimuli, including hypoxia and endothelin. RhoA/Rho kinase-mediated vasoconstriction plays an important role in the pathogenesis of PH in CDH (34).

Vascular endothelial growth factor (VEGF) is an important factor involved in early angiogenesis, endothelial cell proliferation, and differentiation. Angiogenesis is regulated with proangiogenic factors such as VEGF and antiangiogenic proteins including soluble fms-like tyrosinekinase-1 (sFlt-1). Down regulation of VEGF has been related in CDH animal models as a leading cause of the observed abnormal vasculature (35).

In-vitro cell culture, a new evolving technique can provide better insight and understanding of different cell interactions in the pathogenesis of CDH. PPF fibroblast is a type of cell culture that was developed using mouse embryos. It shows that these fibroblasts, maintain the expression of key genes in normal diaphragm development (36). Inducible pluripotent stem cells (iPSC) from fetuses and infants with CDH have been used to establish a reproducible ex vivo model of lung development (37). This in-vitro cell culture system can be a cost-effective method for exploring the underlying mechanism and cellular interactions in CDH. In addition, a better understanding of the disease pathogenesis may lead to an improved investigation of therapeutic targeted interactions.

## Molecular aspect of CDH

CDH is usually a sporadic condition; however, familial cases have been reported (38) An underlying genetic cause has been identified in less than 50% of sporadic and familial cases (29). Among the identifiable genetic causes of CDH, cytogenetic abnormalities and single gene disorders have been described. The most prevalent aneuploidy associated with CDH, trisomy 18, occurs in 2%–5% of CDH (39). Trisomy 13 account for <1% of CDH cases (40). Trisomy 21 is the most frequently occurring aneuploidy identified in children with Morgagni hernia (41).

The significance of copy number variations (*CNVs*) has been related along with the disruption of essential signaling pathways as a causative factor in the development of the diaphragm (26).

It has to be noted that the majority of CNVs identified in patients with CDH are deletions, suggesting that haploinsufficiency could be a crucial mechanism of impaired diaphragm development (26) However, several CDH-related chromosomal loci are affected also by copy number gains (43). The most common *CNVs* associated with CDH include tetrasomy 12p (44), 15q26.1–26.2 deletion, 8p23.1 deletion (*OMIM* 222400) (45), 8q23.1 deletions (46) and 4p16 deletion (also known as Wolf–Hirschhorn syndrome (*OMIM* 194190)) among others (47). The analysis of numerous CDH patients with a chromosome 15q26.2 microdeletion has been particularly crucial, as it revealed a common region of overlap resulting in loss of one *NR2F2* (*COUP*-*TFII*) allele (*OMIM* 142340) (48). This allele is a member of the orphan nuclear receptors and is expressed in regions critical for the formation of the diaphragm during embryonic development (40).

Pathogenic variants in several single genes have been described in both syndromic and non-syndromic cases of CDH. Among the syndromic form of CDH, Cornelia de Lange Syndrome is of particular interest, as the presence of CDH is associated with poor prognosis (42). CDH is also a cardinal feature of Fryns syndrome. The genetic etiology of Fryns syndrome has not been definitively established, although it can be caused by variants in glycosylphosphatidylinositol anchor pathway genes (49).

CDH patients without any other associated birth defects usually manifest with pulmonary hypoplasia of variable degrees. The poor lung growth is not necessarily related to the compression effects of the herniated organs. Indeed, studies have shown that there are primary defects in lung growth that precedes CDH. Disruption in mesenchymal cell function or mutation in the genes encoding transcription factors, molecules involved in cell migration, and extracellular matrix components can all play a complex role in the development of CDH. Mesenchymal cell differentiation abnormalities triggered by environmental or genetic factors can be associated with diaphragmatic or extra diaphragmatic defects as suggested by the mesenchymal hit hypothesis (50). Transcriptional factors which regulate the genes for mesenchymal cell function in the diaphragm, lung, and other organs play an important regulatory function and their deficiencies have been reported with impaired structure integrity, apoptosis, and abnormal cell differentiation (51). *WT*1, *FOG*-2, *TA*-4are the zinc finger transcription factors associated with pulmonary and extrapulmonary manifestation in CDH.

CDH patients without any other associated birth defects usually manifest with pulmonary hypoplasia of variable degree. The poor lung growth is not necessarily related to the compression effects of the herniated organs. Indeed, studies have shown that there are primary defects in lung growth that precede CDH (50, 53).

Identifying an underlying genetic etiology in patients with CDH could significantly impact recurrence risk estimations, mortality rates, and the availability and outcomes of therapy, ultimately improving clinicians' ability to counsel families. Analysis of genetic testing results in one of the largest CDH cohorts published to date provided significant insight into the diagnostic yield of genetic tests in different CDH groups (54). This cohort included 411 patients, 322 with isolated/nonsyndromic CDH and 89 with complex/syndromic CDH. Genetic testing was diagnostic in 57% of infants with complex/syndrome CDH; of them, a causative cytogenetic abnormality was identified in 73% of cases, while a single gene disorder was identified in the other 27% (54). The diagnostic yield of exome sequencing in this group was consistent with previous publications (55). By contrast, genetic testing was diagnostic in only 2% of infants with isolated/nonsyndromic CDH (54). However, it must be noted that most patients in this group were tested only for cytogenetic abnormalities. In addition, for the subjects in which exome sequencing was performed, the indication was developmental delay and/or autism spectrum disorder.

Overall, based on the data available in the literature to date, there is consensus that genetic testing is warranted, especially in individuals with complex/syndromic CDH.

## Metabolic aspect of CDH

Over the last decade, lung metabolism has been extensively studied with the goal of unfolding metabolic dysfunction and association with various diseases (56). However, there are still several aspects that need to be elucidated. In a recent study, significant alterations in antioxidant metabolites, glycolytic energy metabolites, and nucleotide metabolites were associated with CDH in animal models (57). Increased oxidative stress play a role in development of CDH (58). The normal growth of the embryo is sensitive to the balance between antioxidant activity and the production of reactive oxygen species. The endogenous redox state regulates cell proliferation and differentiation during the critical period of active growth *via* signaling pathway (59). Disturbance in this homeostasis can lead to congenital malformations and dysmorphogenesis (60). Hypoxic environment in CDH causes enhanced production of reactive oxygen species (ROS) leading to remodeling of growing vessels *via* smooth muscle cell hyperplasia, endothelial dysfunction, and enhanced contractility that eventually manifests as PPHN (61, 62). A low level of antioxidant activity with high ROS may destroy the signaling pathways to regulate the growth of developing organs (63). Supplementation of Vit E as an antioxidant in rat model have shown some lower trend to CDH but does not have any significant effect on lung hypoplasia (64) Deficient antioxidant activity can be a future therapeutic target for CDH. In addition, a hypoxic environment leads to anaerobic metabolism and subsequent energy failure with inadequate adenosine triphosphate (ATP) production (65). Elevation of lactate and significant depletion of ATP has been described with CDH. Nucleotides are organic molecules consisting of a nucleoside and phosphate. They serve as monomeric units of the nucleic acid polymers-deoxyribonucleic acid (DNA) and ribonucleic acid (RNA) both of which are essential for tissue growth and cell replication. Nucleotides also play a role as coenzymes and messengers in signaling pathways involving cyclic adenosine monophosphate (cAMP) and cyclic guanosine monophosphate (c-GMP). Alteration in nucleotide synthesis, degradation, and imbalance has also been described in CDH but the exact correlation remains unclear. Further, retinoid (Vitamin A), and its metabolites play an important role in fetal growth and development. Vitamin A is an important micronutrient and its deficiency as well as excess have been described in literature as congenital defects and teratogenicity in offspring. Retinoid hypothesis describes abnormal retinoid signaling early in the diaphragm development leading to the development of CDH (66). Dietary deficiency of vitamin A has been described in literature with increased incidence of CDH in animal models but has not been validated in a recent National Birth Defects Prevention Study (67). Although experimental animal data suggest dietary factors including, low intakes of B-vitamins, choline, protein, retinol, and certain minerals in the maternal diet may be associated with CDH among offspring but lacks a clear body of evidence (68). Similarly, pre-conceptional maternal folic acid intake has failed to show any association with the development of CDH (69).

## Conclusions

CDH is a heterogenous disease and multiple factors play a role in its pathogenesis. Understanding the underlying cellular, molecular, and metabolic aspects of CDH is crucial to improve morbidity and mortality. New advances in genetic testing and isolation of genes implicated in CDH development are especially important, as they could represent a very promising target for new therapeutic strategies.
